# Parenting and care: a complex role in the development of mental health

**DOI:** 10.1007/s00787-020-01633-1

**Published:** 2020-09-01

**Authors:** Anna Fuchs, Michael Kaess

**Affiliations:** 1 Centre for Psychosocial Medicine, Department of Child and Adolescent Psychiatry, University Hospital Heidelberg, Heidelberg, Germany; 2 Department of Psychology, Pennsylvania State University, University Park, PA USA; 3 University Hospital of Child and Adolescent Psychiatry and Psychotherapy, University of Bern, Bern, Switzerland

The home environment children grow up in has been of particular interest in developmental psychopathology research due to its importance in interaction with and beyond genetic influences [1]. A healthy and well-functioning home environment supports children’s physical, psychological and socio-emotional development, whereas adverse home environments put children at risk for the development of psychiatric disorders. Most importantly, it is parents’ physiological, behavioral and emotional regulatory capacities that allow them to parent in a healthy way and support children’s normative development [2–4]. If parents’ regulation and positive parenting are compromised, for example due to a parent’s own history of trauma or parental mental disorder, children may not be provided with adequate environmental responses and tools they need for healthy development, which increases the risk for psychiatric disorders [5–8]. For example, experiences of deprivation and threat are both linked with higher levels of psychopathology and higher risk for a variety of psychiatric disorders [9, 10]. Further, higher household chaos and lower socioeconomic status have been associated with lower self-control and higher risk taking as well as poor mental health in children and youth [11, 12]. Interestingly, research further suggests that high-quality childcare matters more for children who come from disadvantaged home environments, demonstrating the compensatory or aggravating potential of out-of-home childcare [13]. Consequently, both home environment and out-of-home care environments should be focused on in clinical research to elucidate appropriate approaches to prevention and intervention.

Research and debate thrive on challenging common assumptions. In this edition, two fairly common assumptions are challenged: First, it is often assumed that a parental history of mental illness should inevitably lead to mental health service utilization in offspring, and second, out-of-home childcare is associated with higher levels of stress including higher cortisol levels in children.

There are a significant number of studies showing that maternal mental disorders are linked with higher rates of disorders in children, with children’s psychiatric risk being at least twofold compared to children whose parents have no history of mental disorders [7]. Parental mental disorder further significantly predicts mental health service utilization [14]. Information about familial transmission of mental disorders rightfully informs treatment decisions in child and adolescent psychiatry. However, as we assume children of mentally ill parents are generally at risk, may this potentially lead to overdiagnosis of mental disorder in those children, provoking overtreatment and misallocation of healthcare resources? As clinical resources are sparse, predictors of overtreatment need to be identified and discussed. Indeed, in their study examining 2317 healthy children without any lifetime diagnosis of mental disorder, Stalujanis and colleagues (2019) report a significantly higher likelihood of service use in healthy children of mentally ill parents compared to offspring of parents without mental disorders [14]. According to these results, children of mentally ill parents may not only be treated for mental disorders, but also for subclinical symptomatology.

Stalujans and colleagues’ findings contribute to two broader ongoing debates: First, should we treat at-risk children and youth to prevent an aggravation of subclinical symptoms into psychiatric disorders [15, 16]? After all, diagnostic decisions are not always without ambiguity, and it is up to the individual clinician to either allocate or deny treatment [17]. The “early intervention” approach that commonly includes treating borderline and at-risk cases certainly provides needed support and may prevent maladaptive development in the long run. However, given limited health service resources, this decision unfortunately always comes at the cost of not treating children who might need resources more urgently. Further, receiving treatment in clinical settings mostly due to parental risk factors may be stigmatizing for children (Corrigan and Miller 2004); as such, potential side effects of early intervention need to be assessed. Parents who have had contact with mental health services themselves have been shown to be less hesitant to seek clinical help for their children [18]. This reported credit of trust could be used for expanded prevention efforts rather than clinical treatment. Higher investments in prevention programs integrated into primary care and school systems, which have shown promising results and effectiveness [19], may help balancing the needs of affected families while circumventing overburdening in clinical settings.

Besides child and adolescent self-reports, parent reports on child symptomatology are a pivotal piece of information in the diagnostic process. The second debate, therefore, raises the issue that, in case of mentally ill parents, perceptions may be biased, and thresholds for problematic behaviors and emotions in children may be lowered [20, 21]. Parental psychopathology has been shown to be associated with higher ratings of psychopathology in children compared to teacher reports or child self-report [22]. This again highlights the importance of cross-informant integration in clinical settings and supports the call for further development of reliable and valid multi-informant clinical tools [23].

Comparison of children’s cortisol levels in at-home and out-of-home care contexts has led to the robust and well-replicated finding that cortisol production differs on childcare days and non-childcare days [24]. Further, this increase in cortisol over the course of the day seems to be especially notable in preschool children [25]. These results have led to a debate about potentially increased stress levels of children in out-of-home care as well as the extent to which these cortisol level increases may negatively affect children's development and whether out-of-home daycare may be too stressful for children [24]. However, as Tervahartiala and colleagues (2019) note, conclusions from these earlier findings are limited, as not one of these studies included an at-home childcare comparison group. Instead, prior studies assessed the same child during their out-of-home childcare day and during their at-home day [26]. In line with prior research, Tervahartiala and colleagues found that in their sample of 213 toddlers, children in out-of-home daycare show increased afternoon cortisol levels on days they did not spend at home. However, contrary to the authors’ expectations, overall cortisol levels were on average 30% higher in the at-home childcare group than in the out-of-home childcare group. Importantly, the increase of afternoon cortisol levels found in the complete sample of 213 children was partly explained by the fact that samples were taken 15–60 min after the children’s daytime naps, which was in turn associated with 46% higher cortisol levels.

These findings have important implications for our understanding of daycare contexts and its potential harm to children. First, children’s sleeping patterns at daycare and at home differ and that seems to play an important role. Cortisol after napping may follow a pattern similar to the cortisol awakening response (CAR) [27]. Especially younger children often have mandatory naptimes that may impact a groups’ afternoon cortisol levels. An earlier study investigating the impact of mandatory naptime in daycare found a similar, albeit not significant, rise in cortisol after the children’s nap [28]. In a different study investigating cortisol in a daycare context, a significant decrease of cortisol immediately after the nap was observed, followed by an increase, again suggesting a pattern comparable to the CAR [29]. Thus, cortisol levels in children may differ significantly not due to environmental stressors but due to differences between nap times in at-home and out-of-home care.

Second, it seems premature to conclude adverse effects of out-of-home childcare based on cortisol assessments without the inclusion of comparison groups into research designs. More research is needed replicating Tervahartiala and colleagues’ findings, using different age ranges and different caregiving contexts. Tervahartiala and colleagues examined children in a very structured and high-quality out-of-home daycare program, whereas no data were analyzed regarding the quality of the at-home care. To be able to discuss the adaptiveness of lower or higher cortisol in different contexts, associations between cortisol levels in at-home and out-of-home caregiving contexts and child development should be investigated longitudinally and include important confounding factors such as caregiving quality and child characteristics [30].

Overall, this issue includes important articles on the role of parenting and care in the development of mental health. However, these articles also illustrate that the relationship between parenting, care and mental health is complex and easy causal conclusions are most likely not warranted at all.

## References

[1] Assary E, Vincent J, Machlitt-Northen S, et al (2020) The role of gene-environment interaction in mental health and susceptibility to the development of psychiatric disorders. In: Beyond our genes. Springer, Berlin, pp 117–138

[2] Bridges RS (2015). Neuroendocrine regulation of maternal behavior. Front Neuroendocrinol.

[3] Feldman R (2012). Parent–infant synchrony: a biobehavioral model of mutual influences in the formation of affiliative bonds. Monogr Soc Res Child Dev.

[4] Hajal NJ, Paley B (2020). Parental emotion and emotion regulation: a critical target of study for research and intervention to promote child emotion socialization. Dev Psychol.

[5] Ahun MN, Consoli A, Pingault J-B (2018). Maternal depression symptoms and internalising problems in the offspring: the role of maternal and family factors. Eur Child Adolesc Psychiatr.

[6] Andreas A, White LO, Sierau S (2018). Like mother like daughter, like father like son? Intergenerational transmission of internalizing symptoms at early school age: a longitudinal study. Eur Child Adolesc Psychiatr.

[7] Johnson SE, Lawrence D, Perales F (2018). Prevalence of mental disorders among children and adolescents of parents with self-reported mental health problems. Community Ment Health J.

[8] Plant DT, Pawlby S, Pariante CM, Jones FW (2017). When one childhood meets another–maternal childhood trauma and offspring child psychopathology: a systematic review. Clin Child Psychol Psychiatr.

[9] Carr CP, Martins CMS, Stingel AM (2013). The role of early life stress in adult psychiatric disorders: a systematic review according to childhood trauma subtypes. J Nerv Ment Dis.

[10] Miller AB, Sheridan MA, Hanson JL (2018). Dimensions of deprivation and threat, psychopathology, and potential mediators: a multi-year longitudinal analysis. J Abnorm Psychol.

[11] Herrmann J, Vogel M, Pietzner D (2018). Factors associated with the emotional health of children: high family income as a protective factor. Eur Child Adolesc Psychiatr.

[12] Holmes C, Brieant A, Kahn R (2019). Structural home environment effects on developmental trajectories of self-control and adolescent risk taking. J Youth Adolesc.

[13] Watamura SE, Phillips DA, Morrissey TW (2011). Double jeopardy: Poorer social-emotional outcomes for children in the NICHD SECCYD experiencing home and child-care environments that confer risk. Child Dev.

[14] Stalujanis E, Meinlschmidt G, Belardi A, Tegethoff M (2019). Maternal psychopathology and offspring mental health service utilization in adolescents without mental disorders: a national representative survey. Eur Child Adolesc Psychiatr.

[15] Bijl RV, de Graaf R, Hiripi E (2003). The prevalence of treated and untreated mental disorders in five countries. Health Aff (Millwood).

[16] Kessler RC, Merikangas KR, Berglund P (2003). Mild disorders should not be eliminated from the DSM-V. Arch Gen Psychiatr.

[17] Merten EC, Cwik JC, Margraf J, Schneider S (2017). Overdiagnosis of mental disorders in children and adolescents (in developed countries). Child Adolesc Psychiatr Ment Health.

[18] Owens PL, Hoagwood K, Horwitz SM (2002). Barriers to children’s mental health services. J Am Acad Child Adolesc Psychiatr.

[19] Beardslee WR, Gladstone TR, O’Connor EE (2011). Transmission and prevention of mood disorders among children of affectively ill parents: a review. J Am Acad Child Adolesc Psychiatr.

[20] Seiffge-Krenke I, Kollmar F (1998). Discrepancies between mothers’ and fathers’ perceptions of sons’ and daughters’ problem behaviour: a longitudinal analysis of parent–adolescent agreement on internalising and externalising problem behaviour. J Child Psychol Psychiatr.

[21] Teagle SE (2002). Parental problem recognition and child mental health service use. Ment Health Serv Res.

[22] De Los RA, Kazdin AE (2005). Informant discrepancies in the assessment of childhood psychopathology: a critical review, theoretical framework, and recommendations for further study. Psychol Bull.

[23] Martel MM, Markon K, Smith GT (2017). Research review: Multi-informant integration in child and adolescent psychopathology diagnosis. J Child Psychol Psychiatr.

[24] Vermeer HJ, van IJzendoorn MH (2006). Children’s elevated cortisol levels at daycare: a review and meta-analysis. Early Child Res Q.

[25] Bernard K, Dozier M, Bick J, Gordon MK (2015). Intervening to enhance cortisol regulation among children at risk for neglect: results of a randomized clinical trial. Dev Psychopathol.

[26] Tervahartiala K, Karlsson L, Pelto J (2019). Toddlers’ diurnal cortisol levels affected by out-of-home, center-based childcare and at-home, guardian-supervised childcare: comparison between different caregiving contexts. Eur Child Adolesc Psychiatr.

[27] Schlarb AA, Lollies F, Claßen M (2016). Cortisol and sleep in infancy and early childhood. Somnologie.

[28] Thorpe KJ, Pattinson CL, Smith SS, Staton SL (2018). Mandatory naptimes in childcare do not reduce children’s cortisol levels. Sci Rep.

[29] Watamura SE, Sebanc AM, Gunnar MR (2002). Rising cortisol at childcare: Relations with nap, rest, and temperament. Dev Psychobiol.

[30] Gunnar MR, Kryzer E, Van Ryzin MJ, Phillips DA (2010). The rise in cortisol in family day care: Associations with aspects of care quality, child behavior, and child sex. Child Dev.

